# Age-related decreased inhibitory vs. excitatory gene expression in the adult autistic brain

**DOI:** 10.3389/fnins.2014.00394

**Published:** 2014-12-08

**Authors:** Louie N. van de Lagemaat, Bonnie Nijhof, Daniëlle G. M. Bosch, Mahdokht Kohansal-Nodehi, Shivakumar Keerthikumar, J. Alexander Heimel

**Affiliations:** ^1^Centre for Neuroregeneration and Centre for Clinical Brain Sciences, University of EdinburghEdinburgh, UK; ^2^Department of Human Genetics, Radboud University Medical CenterNijmegen, Netherlands; ^3^Bartiméus, Institute for the Visually ImpairedZeist, Netherlands; ^4^Radboud Institute for Molecular Life Science, Radboud University Medical CenterNijmegen, Netherlands; ^5^Donders Institute for Brain, Cognition and Behavior, Radboud University Medical CenterNijmegen, Netherlands; ^6^Department of Neurobiology, Max Planck Institute for Biophysical ChemistryGöttingen, Germany; ^7^Department of Biochemistry, La Trobe Institute for Molecular Science 1, La Trobe UniversityMelbourne, VIC, Australia; ^8^Cortical Structure and Function Group, Netherlands Institute for NeuroscienceAmsterdam, Netherlands

**Keywords:** autism, excitation, inhibition, balance, gene expression, age effect

## Abstract

Autism spectrum disorders (ASDs) are neurodevelopmental disorders characterized by impaired social interaction and communication, and restricted behavior and interests. A disruption in the balance of excitatory and inhibitory neurotransmission has been hypothesized to underlie these disorders. Here we demonstrate that genes of both pathways are affected by ASD, and that gene expression of inhibitory and excitatory genes is altered in the cerebral cortex of adult but not younger autistic individuals. We have developed a measure for the difference in the level of excitation and inhibition based on gene expression and observe that in this measure inhibition is decreased relative to excitation in adult ASD compared to control. This difference was undetectable in young autistic brains. Given that many psychiatric features of autism are already present at an early age, this suggests that the observed imbalance in gene expression is an aging phenomenon in ASD rather than its underlying cause.

## Introduction

Autism spectrum disorders (ASDs) are neurodevelopmental disorders with impairments in social interaction, language or behavior, and repetitive behavior or restricted interest. In the United States 1 in 68 children has an ASD (Centers for Disease Control and Prevention (US) and Centers for Disease Control and Prevention (US) Epidemiology Program Office, 2014). ASDs are highly heritable, yet currently only 20% of the cases have an identified molecular cause (Rosti et al., 2014), suggesting a multigenic mode of inheritance for autism. This is consistent with the popular hypothesis that ASDs result from an increased ratio of excitatory/inhibitory (E-I) neurotransmission, with many variants and mutations of different genes leading to a disturbed E-I balance (Rubenstein and Merzenich, 2003). In support of this hypothesis, several lines of evidence point to alterations in molecular components of inhibitory synapses leading to ASD (Hussman, 2001; Baroncelli et al., 2011). For example, duplications on chromosome 15q11-q13, encompassing *GABRB3*, *GABRA5*, and *GABRG3* are associated with autism (Menold et al., 2001) and reduced expression of several genes involved in central inhibitory synapses of ASD individuals has been reported. Post-mortem brain tissue showed a reduction of the inhibitory GABA_A_ and GABA_B_ receptors in autistic individuals (Blatt et al., 2001; Fatemi et al., 2009a,b, 2014; Oblak et al., 2009, 2010) as well as a reduction of levels of GAD65 and GAD67, the enzymes that are necessary to produce the inhibitory neurotransmitter GABA (Fatemi et al., 2002).

Whilst several studies implicate decreased inhibition in autism, alterations in excitatory synapses in ASD are less known. The original genetic evidence for an excitatory role in the disturbed E-I balance hypothesis was an association study identifying the ionotropic glutamate receptor GRIK2, also known as GluR6 (Jamain et al., 2002; Rubenstein and Merzenich, 2003). This association was, however, not present in a later Indian population study (Dutta et al., 2007). Furthermore, in addition to an excitatory role, GRIK2 has been shown to depress inhibitory synaptic transmission (Chergui et al., 2000). There has been little further direct physiological evidence supporting the idea of disturbed excitatory transmission in ASD. In blood, an increased level of the excitatory neurotransmitter glutamate was identified (Shinohe et al., 2006), but the mechanisms underlying this observation are far from clear. Using proton magnetic resonance imaging, both increased and decreased levels of glutamate/glutamine have been measured in different regions of the brains of ASD individuals (Page et al., 2006; Bernardi et al., 2011; Horder et al., 2013), but again it is unclear how this might affect the pathophysiology of autism.

The popularity of the E-I imbalance hypothesis in the absence of much direct evidence for a role of excitation prompted us to attempt to quantify the relative strength of excitation and inhibition in ASD patients and controls. First, we identified a list of genes impacting excitatory or inhibitory neurotransmission and checked this list for associations with ASD. We then used the mean expression levels of the two sets of genes as proxy measures for excitation (E) and inhibition (I) and their difference E-I. We computed these measures for the samples from two publicly available ASD and control microarray datasets derived from cortical brain tissue (Voineagu et al., 2011; Chow et al., 2012). Although these datasets differ in age of individuals and precise cortical tissue location, the findings from both studies agree, pointing to a broader mechanism involved in the pathophysiology of autism.

## Materials and methods

### Marker selection

Marker genes for excitatory vs. inhibitory synapses were selected by retrieving the human genes associated with specific gene ontology (GO, version of September, 2013) terms from amigo.geneontology.org (Table 1). We classified GO terms as related to excitation based on proteins being present in excitatory synapses or having a role in amplifying excitatory neurotransmission or decreasing inhibitory neurotransmission. Conversely, GO terms describing presence in inhibitory synapses, attenuation of excitatory transmission or a tendency to increase inhibitory neurotransmission were classified as related to inhibition. Thus, the marker selection was based on protein function, blind to any downstream analysis. Genes classed as having both excitatory and inhibitory functions were removed from consideration.

**Table 1:** **Blind marker selection based on Gene Ontology terms**.

**GO term (ID)**	**Gene symbols**
Excitatory synapse (GO:0060076)	ACTR3; DLG4; ELFN1; FGFR2; GRIN1; LRRTM1; LRRTM2; NETO1; NLGN3; NTRK2; SHANK1; SLC17A7; SRPX2; SYNDIG1; SYP; SYT1
Positive regulation of synaptic transmission, glutamatergic (GO:0051968)	ADCYAP1; DRD1; EGFR; GLUL; NRXN1; NTRK1; NTRK2; PTGS2; RELN; SHANK3; TNR1
Positive regulation of excitatory postsynaptic membrane potential (GO:2000463)	DRD4; GRIN1; NRXN1; PRKCZ; RELN; SHANK1; SHANK3
Negative regulation of synaptic transmission, GABAergic (GO:0032229)	ADRA1A; NPY5R; SLC6A1; STXBP1
Glutamate biosynthetic process (GO:0006537)	GLUD1; GLUD2; GLS; PRODH; PRODH2
Inhibitory synapse (GO:0060077)	CEP112; GABRG2; IQSEC3; MAF1; SLC32A1
Positive regulation of synaptic transmission, GABAergic (GO:0032230)	GRIK1; PRKCE; TAC1; TACR1
Negative regulation of excitatory postsynaptic membrane potential (GO:0090394)	CELF4; MTMR2; NPY2R; S1PR2
Negative regulation of synaptic transmission, glutamatergic (GO:0051967)	ATAD1; DRD2; GRIK1; GRIK2; GRIK3; HTR2A; NPY2R; PLA2G6
Gamma-aminobutyric acid biosynthetic process (GO:0009449)	GAD1
Regulation of synaptic transmission, GABAergic (GO:0032228)	NF1

### Analysis of literature reports of large scale mutation studies

Large-scale CNV and exome sequencing studies of autism were identified (Pinto et al., 2010; Neale et al., 2012; O'Roak et al., 2012; Sanders et al., 2012). Copy number variants and *de novo* protein sequence variants were identified in supplementary information from these studies and compared with blind-chosen excitatory and inhibitory markers based on gene symbol. An additional mutation study (Prasad et al., 2012), was found in the Simons Foundation Autism Research Initiative (SFARI) GENE database (Basu et al., 2009) located at www.sfari.org/resources/sfari-base.

### Microarray data processing

#### Voineagu dataset preprocessing

Raw microarray data from autistic and control cortical samples were downloaded from GEO, accession number GSE28521, normalized and corrected using the lumiR and lumiExpresso functions from the lumi package in R. Expression values for each sample were extracted. Then correlations were computed between all pairs of samples; several samples were excluded based on the fact that they were poorly correlated with a replicate sample from the same individual and also poorly correlated with other individuals. Samples thus excluded were A_AN09730_T, A_AN17138_T, A_AN09730_F, A_AN17777_T, C_AN00142_T, C_AN04479_T, and A_AN17138_F. Remaining replicate samples were averaged within an individual. This left 30 samples, 14 with autism and 16 controls. Mature samples of age 20+ years, composed of 9 cases and 13 controls, were analyzed for excitatory and inhibitory gene expression, as well as E-I difference, as described below. Eighty per cent power to detect a significant result using a *t*-test is achieved for mature samples at the α = 0.05 level at a standardized mean difference (Cohen's *d*) of 1.3 standard deviations.

#### Chow dataset preprocessing

Processed cortical data per array were downloaded from GEO, accession number GSE28475, and averaged signals were plotted, all vs. all. Inspection of correlation plots between samples revealed a number of frozen DASL samples with poor correlation with most or all other samples: 15_1, 20_1, 31_1, 32_1, 45_1, 46_1, 49_1, 50_1, 51_1, 52_1, 57_1, 57_2, 61_1, 64_1, 69_1, 70_1, and 74_1. After exclusion of these samples, two samples, 22_1 and 23_1, showed good correlation with all remaining samples, so all samples were quantile normalized to the combination of these samples. Correlations were then computed between all pairs of samples, revealing Pearson correlation coefficients greater than 0.9 for 41 brains, 22 control and 19 autistic. Mature samples of age 20+ years, composed of 6 cases and 7 controls, were assessed for excitation, inhibition, and E-I difference. Eighty per cent power to detect a significant result using a *t*-test is achieved for mature samples at the α = 0.05 level at a standardized mean difference (Cohen's *d*) of 1.9 standard deviations. Secondarily, the same analysis was performed on immature samples, younger than 15 years, composed of 11 cases and 11 controls. Eighty per cent power to detect a significant result using a *t*-test is achieved for immature samples at the α = 0.05 level at a standardized mean difference (Cohen's *d*) of 1.2 standard deviations.

#### Calculation of E and I

Probes for genes with excitatory and inhibitory effect were identified and those targeted to all splice forms or the only splice form were retained. This left at least one probe per gene that could be analyzed. If there were multiple cortical samples from one brain, then they were averaged within a brain. Expression values for each probe were then shifted and scaled to a range [0,1]. Scaled and shifted expression values for genes for which there were multiple probes were then averaged across probes. Finally, these scaled and shifted expression values for excitatory and inhibitory type genes were averaged to obtain E, I and E-I values for each brain. Expression values for each probe in both datasets are provided in Supplementary Data Sheet 1.

#### Chow dataset regression analysis with respect to age

Regression analysis of expression to deduce age effects was carried out as follows. Non-linear least squares regression (nls function in R) was used to fit a regression model; in that model a set of *n_case_* + *n_control_* expression values were fitted to a regression model described by Expression = A1 + B1^*^age + A2^*^weights + B2^*^weights^*^age. The *n_case_* + *n_control_* weights were zero for controls and 1.0 for cases. The principal outputs of the model were the parameters A1, B1, A2, and B2 and their standard errors.

We computed power to detect a correlation difference between autism and control using the same models as used in our computations. We assigned cases and controls simulated uniform random ages between 0 and 50 years. Correlation was simulated in 19 autistic samples and no correlation was simulated in 22 normal samples. Simulated correlations were in the range 0 ≤ R < 0.86 with 1000 replicates at each of 25 correlation levels. Eighty per cent power to detect significant difference in correlation at the α = 0.05 level was achieved at Pearson *r* = 0.81.

#### Chow dataset regression analysis with respect to nervous system tissues

Regression analysis of expression was carried out using the same method as described above, except that we analyzed group averages of markers of neuronal, astrocytic, oligodendrocytic, and microglial cell populations. The normalized and then averaged expression in each of the four tissues was compared to age in cases and controls using the same non-linear model as described above. Marker sets, microarray probe identifiers, and fitted model parameters are found in Supplementary Table 1.

#### Mixed analysis of age and neuronal markers to explain excitatory and inhibitory gene expression in the Chow dataset

The concern arose that observed age-related changes in excitatory and inhibitory gene expression in cases compared to controls would be a reflection of changes in nervous system cell populations; this concern focussed particularly on neurons. To address this, the neuronal cell population was represented by an average of neuron-specific markers (described above). Two terms related to neurons were added to the age model, neuronal expression in controls and difference in neuronal expression between cases and controls. To limit the number of parameters fitted and minimize overfitting, the expression intercept (at age 0) in cases was assumed to be the same as in controls and omitted. As before, weights were assigned zero for controls and one for cases. This resulted in a model with five parameters fitted to 41 data points: Expression = A1 + B1^*^age + B2^*^weights^*^age + C1^*^neurons + C2^*^weights^*^neurons. Results of this analysis are in Supplementary Table 1.

## Results

### Marker selection

We selected two groups of genes, termed excitatory and inhibitory as described in methods, based on a selection of gene ontology (GO) terms. In principle, excitatory genes were considered to amplify excitatory (glutamatergic) neurotransmission or attenuate inhibitory (GABAergic) neurotransmission, whilst inhibitory genes were considered to amplify inhibitory neurotransmission or attenuate excitatory neurotransmission (Table 1). Genes classed as having both excitatory and inhibitory functions were dropped from our analysis. This led to a list of 38 excitatory genes and 21 inhibitory genes.

Components of the excitatory postsynaptic density derived from human cortex (Bayes et al., 2011) were overrepresented in our excitatory gene set (6.1 fold enrichment, *p* < 10^−8^, hypergeometric test) relative to the human genome background. Our inhibitory gene set was not significantly enriched with postsynaptic density genes (1.6 fold enrichment, *p* = 0.13, hypergeometric test).

### Mutations of excitatory and inhibitory genes in ASD individuals

We first asked to what extent known copy number variants (CNVs) or damaging protein sequence variants were known for our set of excitatory and inhibitory genes. A detailed examination of literature reports of large scale studies of copy number variation and protein sequence variants (Pinto et al., 2010; Neale et al., 2012; O'Roak et al., 2012; Sanders et al., 2012) revealed that some of these genes are affected by mutation in autism. Examination of the public database SFARI GENE (Basu et al., 2009) showed additional mutations identified in autistic individuals only (Prasad et al., 2012). Eight mutations likely of damaging impact affected five excitatory genes and two putatively damaging mutations impacted two inhibitory genes (Table 2). We used mutations from cases only to compute a measure of enrichment of mutations in this gene set in autism using Poisson statistics; compared to 3281 total gene hits in autism, 10 putatively damaging hits in this set of 59 genes do not reflect significant enrichment (1.2 fold; *p* = 0.19, Poisson test). Focussing only on the likely-damaging protein modifications from the studies of O'Roak et al. (2012), Sanders et al. (2012), and Neale et al. (2012), we find 450 in the genome in autism; compared to this, four hits in our gene set reflect a nominally significant enrichment (3.6 fold; *p* = 0.006, Poisson test). These preliminary findings are encouraging and could motivate the investigation of this gene set in larger case-control association studies, as has been done with other gene sets (Kirov et al., 2012).

**Table 2:** **Known mutations in ASD for excitatory and inhibitory genes**.

**Gene type**	**Symbol**	**Mutation (residue or genome coordinates, hg18)**	**Damaging?**	**Condition**	**Reference**
Excitatory	ELFN1	chr7:1754k-1774k	Loss, exonic	Autism	SFARI, Prasad et al., 2012
Excitatory	GRIK2	chr6:101961k-102006k	Gain, intronic	Autism	SFARI, Prasad et al., 2012
Excitatory	GRIK2	chr6:102425k-102437k	Loss, intronic	Autism	SFARI, Prasad et al., 2012
Excitatory	NRXN1	chr2:50722k-50730k	Loss, exonic	Autism	SFARI, Prasad et al., 2012
Excitatory	NRXN1	chr2:50708k-50721k	Loss, exonic	Autism	SFARI, Prasad et al., 2012
Excitatory	NRXN1	chr2:50421k-50908k	Loss, exonic	Autism	SFARI, Prasad et al., 2012
Excitatory	NRXN1	chr2:50722k-50730k	Loss, intronic	Autism	SFARI, Prasad et al., 2012
Excitatory	NRXN1	chr2:50913k-50958k	Loss, intronic	Autism	SFARI, Prasad et al., 2012
Excitatory	NRXN1	chr2:51045k-51127k	Loss, exonic	Autism	SFARI, Prasad et al., 2012
Excitatory	RELN	p.Q417*	Nonsense	Autism	Neale et al., 2012
Excitatory	SLC6A1	p.A288V	Missense	Autism	Sanders et al., 2012
Excitatory	STXBP1	p.R551C	Missense	Autism	Neale et al., 2012
Inhibitory	CEP112	chr:1761133k-61139k	Loss, intronic	autism	SFARI, Prasad et al., 2012
Inhibitory	GAD1	-	Silent	Autism	O'Roak et al., 2012
Inhibitory	MTMR2	p.N283D	Damaging	Autism	Sanders et al., 2012
Inhibitory	NF1	p.H2459N	Missense predicted tolerated, benign	Autism	Sanders et al., 2012
Inhibitory	NF1	p.A2644V	Missense, predicted probably damaging	Control	Sanders et al., 2012
Inhibitory	NF1	chr17:26493k-26507k	Loss, exonic	Autism	SFARI, Prasad et al., 2012

### Expression of excitatory and inhibitory genes is altered in autism

To gauge the impact of autism on excitatory and inhibitory transmission, we considered two available studies which addressed gene expression in autism in the human cortex (Voineagu et al., 2011; Chow et al., 2012). We used cortical samples from both studies; cortical samples in the Voineagu dataset, though heterogeneous, clustered strongly with other cortical samples irrespective of sample location (Supplementary Figure 1). Discarding cerebellar samples and averaging cortical samples per individual in both datasets results in high correlation across all genes between sample pairs (Supplementary Figures 2, 3).

**Figure 1:**
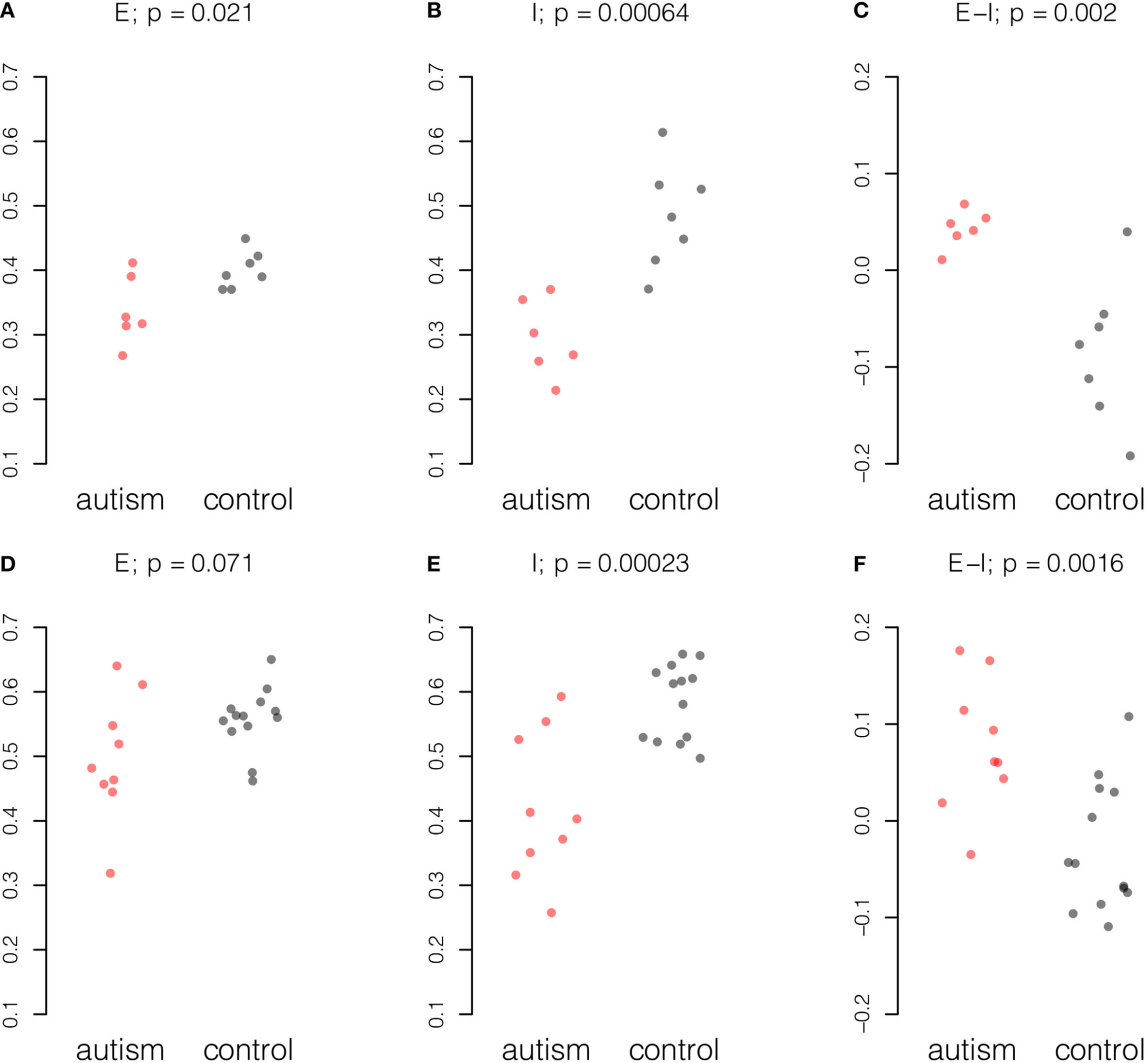
**Summary of excitatory and inhibitory changes in cortical samples from mature individuals**. Expression for each probe scaled to the range [0, 1] and averaged across probes and individuals. Upper panel, six autism and seven control samples in the Chow dataset: **(A)** Excitatory expression, **(B)** Inhibitory expression, and **(C)** Excitatory-inhibitory difference. Lower panel, nine autism and 13 control samples in the Voineagu dataset. **(D)** Excitatory expression, **(E)** Inhibitory expression, and **(F)** Excitatory-inhibitory difference. *P*-values were computed by Student *t*-test.

Gene expression values from 38 excitatory genes and 21 inhibitory genes from each sample were normalized and averaged amongst probes for the same gene and amongst samples from the same individual (see Materials and Methods). Stratifying samples by age of the donor and considering only samples from donors of 20 years of age and older, we found concerted changes in the E-I measure in the data from both studies (Figure 1). A reduction in inhibitory gene expression appears to underpin the change in the balance of excitation and inhibition in both studies. To a smaller extent, the Chow dataset also showed reduction in excitatory expression in mature autistic samples.

### Expression differences in mature autistic brain are age-related

**Figure 2:**
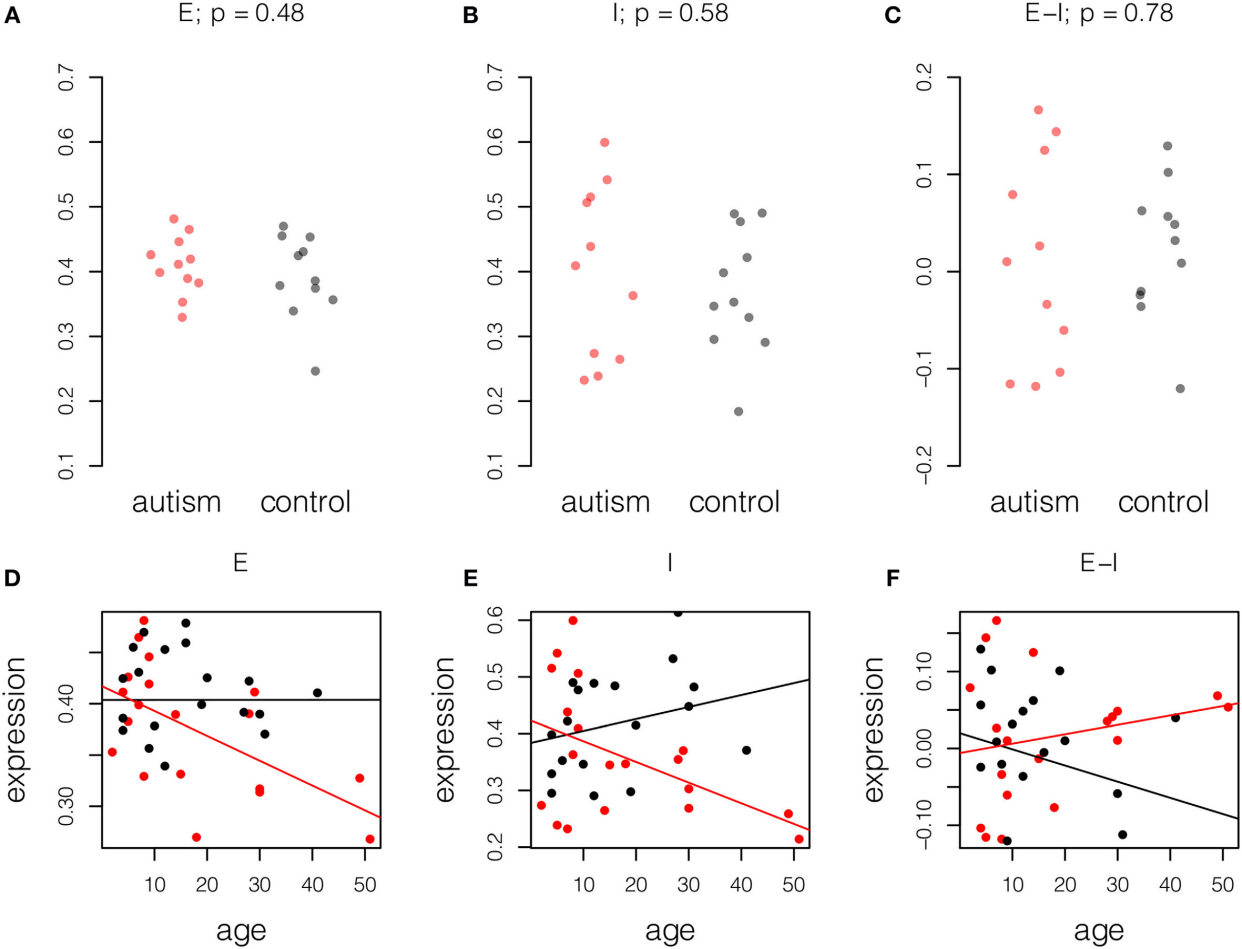
**Age-related changes in excitation and inhibition in autism**. Expression for each probe scaled to the range [0, 1] and averaged across probes and individuals. Upper panel, samples of age less than 15 years, 11 autism and 11 control. *P*-values computed by Student *t*-test. **(A)** Excitatory expression, **(B)** Inhibitory expression, and **(C)** Excitatory-inhibitory difference. Lower panel, fitted models of excitatory and inhibitory molecule expression with age in years in autism cases (red) and controls (black). Model parameters are given ± standard error. **(D)** Excitatory expression does not change with age in controls (fitted model equation: 0.40[±0.02] + 0.0[±7.4]^*^10^−4^^*^ age). However, relative reduction in excitatory expression with age is significant in autistic samples (fitted model equation: 0.014[±0.026] – 0.0024[±0.0011] ^*^ age). **(E)** Inhibitory expression does not change significantly with age in controls (fitted model equation: 0.38[±0.04] + 0.0021[±0.0015] ^*^ age). However, relative reduction in inhibitory expression with age is significant in autistic samples (fitted model equation: 0.039[±0.054] – 0.0057[±0.0023] ^*^ age). **(F)** Difference between excitation and inhibition does not decrease significantly with age in controls (fitted model equation: 0.020[±0.031] – 0.0021[±0.0012] ^*^ age). Relative to controls, increase in E-I difference is also not significant (fitted model equation: −0.026[±0.044] + 0.0033[±0.0019] ^*^ age). Thus, decreases in excitation and inhibition largely cancel in this small dataset.

Given the observation that mature autistic brains show changes in the balance of excitation and inhibition in mature samples, we asked if this condition was life-long or age-related; some expression changes in the aging autistic brain were reported by Chow et al. (2012). We therefore performed the same analysis on a younger set of Chow samples, aged less than 15 years, 11 with autism and 11 controls. In contrast to the mature samples, in which differences in excitation and inhibition were noted, younger samples showed no difference in excitation and inhibition relative to normal samples (Figure 2, upper panel).

To better understand the effect of age on excitation and inhibition in autism, we built combined linear models of expression in the full set of 41 high-quality samples from Chow et al. (19 cases, 22 controls). This was done by fitting a unified least squares model to the data. Control data were modeled as having a linear trend and autistic samples were modeled as having the same trend, plus a difference in trend. This permitted us to assess the significance of the difference between the age effects in cases and controls (Figure 2, lower panel; Table 3). This analysis showed that both excitatory and inhibitory gene expression differ little between autistic and control individuals at birth (E: *p* = 0.6; I: *p* = 0.5). However, a decay in excitatory gene expression of 2.4% per decade of life in cases compared to controls was observed (model coefficient *p* = 0.04). A more severe relative decrease of inhibition of 7.8% per decade was observed in inhibitory expression (model coefficient *p* = 0.02). Taken together, these decays in excitation and inhibition resulted in a general increase in net excitatory expression in autism that failed to significantly diverge from the trend in aging controls (5.4% per decade of life, *p* = 0.08).

**Table 3:** **Fitted model values from regression analysis of neuronal markers and age in the Chow dataset**.

**Data type**	**Model parameter**	**Parameter value ± SE**	***p*-value**
Excitatory	Intercept	0.21±0.03	< 10^−8^
	Age in controls	−0.000045±0.00049	0.9
	Age, case-control difference	−0.0011±0.0007	0.1
	Neurons in controls	0.0018±0.0003	< 10^−6^
	Neurons, case-control difference	0.00012±0.00016	0.5
Inhibitory	Intercept	−0.055±0.048	0.3
	Age in controls	0.0020±0.0008	0.018
	Age, case-control difference	−0.0027±0.0011	0.026
	Neurons in controls	0.0042±0.0004	< 10^−10^
	Neurons, case-control difference	0.00034±0.00026	0.2
Excitatory-	Intercept	0.27±0.06	< 10^−4^
Inhibitory	Age in controls	−0.0020±0.0010	0.049
	Age, case-control difference	0.0015±0.0014	0.3
	Neurons in controls	−0.0024±0.0006	< 10^−3^
	Neurons, case-control difference	−0.00023±0.00033	0.5

### Decline of inhibition with age is not explained by decline in nervous system tissues

We tested whether our expression changes with age could be attributed to generalized decreases in brain tissue with age. We therefore isolated sets of markers and relevant probes for neuronal and glial tissues, following those identified by Kuhn et al. (2011). Identical to our regression analysis of excitatory and inhibitory markers, we analyzed the averaged expression of groups of cell-type markers for neurons (*NEFL*, *ENO2*, *SLC12A5*, *KCNQ2*, and *SCN3A*), astrocytes (*GFAP*, *AQP4*, and *GJA1*), oligodendrocytes (*MOG*, *MAG*, *MOBP*, and *MBP*), and microglia (*CD37* and *CD53*). We found no age differences between cases and controls in any of the groups of cell-type markers (Supplementary Figure 4 and Supplementary Table 1). Whilst in neurons there may be a difference between cases and controls, our sample size is too low to demonstrate this conclusively.

A more robust test for our excitatory and inhibitory expression patterns involves regressing out age as well as the neuronal cell markers mentioned above in cases relative to controls. With respect to neurons, our method is an implementation of so-called Population Specific Expression Analysis (PSEA) (Kuhn et al., 2011). Beyond PSEA, however, our analysis puts the expression of neurons in competition with age to explain variation in gene expression in cases and controls (see Materials and Methods). If decreases in neurons are the sole explanation for the excitatory and inhibitory signals that we observe, this should leave insignificant additional age effects (Table 3). In excitatory markers, we found a slight remaining extra negative trend with age in cases, but this was not significant. A larger sample size would be required to confirm this finding. However, in inhibitory markers, there was a remaining extra negative trend with age in cases, and this was nominally significant (Table 3). Subtracting E-I, there is no significant difference between case and control samples, as before. In other words, the general trends we report survive, even after accounting for changes in broad expression of neuronal markers.

## Discussion

We have conducted a study of excitatory and inhibitory gene expression in autistic individuals, compared with normal individuals. Our classification of genes was obtained by the blind choice of genes based on their annotated functions in neurotransmission. Genes coding for proteins that tend to amplify excitatory neurotransmission or attenuate inhibitory neurotransmission were classified as excitatory. Conversely, those that amplify inhibitory or attenuate excitatory neurotransmission were classified as inhibitory. Genes annotated as participating in both were excluded. Putatively damaging protein-altering mutations in autism showed enrichment in this gene set.

Our study finds excitatory/inhibitory imbalance in ASD, but, based on gene expression evidence, this imbalance only appears and progresses with age. A literature survey revealed mutations in ASD in both the excitatory and inhibitory gene sets. When we calculated a measure for the balance of excitation and inhibition, based on gene expression of the two classes, we found, using a *t*-test, that the balance is shifted toward less inhibition or more excitation in the cerebral cortex of adult autistic individuals in two independent datasets, whereas this difference is not found in immature individuals in the Chow dataset. Unfortunately, there were insufficient immature samples in the Voineagu dataset to retest this finding there.

By further modeling inhibitory gene expression as a linear functions of age in cases and controls in the Chow dataset, we showed a significant trend toward reductions in inhibition with age in cases relative to controls, in keeping with the *t*-test result. Further modeling excitatory gene expression and E-I differences as linear functions of age in cases and controls, these results at first glance appear not to confirm the *t*-test results. However, to make sense of these apparent discrepancies, it must be kept in mind that a *t*-test for difference between cases and controls tests only one parameter; the fitted linear model tests multiple parameters simultaneously, resulting in less-robust parameter fits. In light of this, it makes most sense to postulate, based on this dataset, reductions in both excitatory and inhibitory gene expression with age in cases compared to controls, but that the reductions of inhibitory expression are more pronounced.

A concern arose that the changes in excitatory and inhibitory gene expression we observe are merely a reflection of broader changes in nervous system tissues, and especially neurons; however, introduction of neuronal marker expression to explain age-related changes in inhibitory gene expression resulted in retention of the nominally significant decrease in inhibition with age.

Much of the previous evidence supporting the imbalance hypothesis consisted of separate studies reporting on a select number of genes tested in a select number of brain regions. While it was already clear that the expression of several genes related to GABA-ergic signaling is reduced in autism (Blatt et al., 2001; Fatemi et al., 2002, 2009a,b; Oblak et al., 2009, 2010), it was yet not clear that this was a consistent finding for the GABA-ergic pathway as a whole. Previous reports on reduced inhibitory markers primarily used data from adult brains. The Chow et al. (2012) dataset, whilst of low power, allowed us to study the imbalance in a group of younger individuals. We found no evidence of any imbalance early in life, whereas the increase in E-I associated with autism appears to develop with age. As autism can already be diagnosed before the age of 3 years, this suggests that the E-I imbalance only develops in response to other changes underlying ASD, rather than causing ASD. Greater numbers of samples of a variety of ages will be necessary to confirm this.

We observe two caveats regarding the results we report here. The first caveat is that we quantify excitation-inhibition balance by a single summary value per individual, based on samples derived from a large brain region; this E-I value is based, not on a functional measure of inhibition or excitation, but on mRNA expression levels. Presumably both excitation and inhibition are highly complex processes working differently at different locations, on different timescales, and in different contexts. Obviously, the single value per individual is to a greater or lesser extent insensitive to the complex architecture of excitation and inhibition. Yet, confirmation that the E-I measure is significantly reduced in mature individuals in two separate data sets suggests that a fundamental component of the excitation-inhibition balance can be captured this way. Whilst it is possible to alter the physiological balance of excitation and inhibition using drugs like diazepam, ASD has been resistant to drug treatment and is thus not identical to an acute excitation and inhibition imbalance.

Second, as mentioned above, our findings are based on a small number of samples. This is a reflection of the paucity of publicly available brain expression data from autism samples. Larger sample sizes will be required to improve our knowledge of how autistic brains are different than controls and how the disorder evolves during life. Hypothesis-free approaches, which involve sifting through raw expression data, as well as hypothesis driven approaches like ours, are key elements of an overall progress to understanding. Such studies as this one can inform future study designs with respect to experimental power.

In conclusion, our methods demonstrate usefulness for study of the E-I balance in tissues derived from specific brain regions or other disorders such as Rett syndrome, Angelman syndrome and schizophrenia for which an E-I imbalance is hypothesized (Dani et al., 2005; Fernandez and Garner, 2007; Gonzalez-Burgos et al., 2011; Wallace et al., 2012). Insight into the mechanisms that underlie neurodevelopmental disorders, especially those with implications for broad mechanisms of neurotransmission as we have analyzed here, might serve as a starting point for therapeutic intervention.

### Conflict of interest statement

The authors declare that the research was conducted in the absence of any commercial or financial relationships that could be construed as a potential conflict of interest.
