# Supplementary material for: Age-related decreased inhibitory vs. excitatory gene expression in the adult autistic brain
Source: Front Neurosci. 2014 Dec 8;8:394. doi: 10.3389/fnins.2014.00394 (PMC4259106; doi:10.3389/fnins.2014.00394)
Supplement: Supplementary file 2 [file DataSheet2.DOCX]

***Supplementary Material***

**Age-related decreased inhibitory versus excitatory gene expression in the adult autistic brain**

**Louie N. van de Lagemaat^1*^, Bonnie Nijhof^2†^, Danielle G.M. Bosch^2,3,4,5†^, Mahdokht Kohansal-Nodehi^6†^, Shivakumar Keerthikumar^7†^, J. Alexander Heimel^8^**

† authors contributed equally

^1^Centre for Neuroregeneration and Centre for Clinical Brain Sciences, University of Edinburgh, Edinburgh, United Kingdom.

^2^Department of Human Genetics, Radboud university medical center, Nijmegen, the Netherlands.

^3^Bartiméus, Institute for the Visually Impaired, Zeist, the Netherlands

^4^Radboud Institute for Molecular Life Science, Radboud university medical center, Nijmegen, the Netherlands

^5^Donders Institute for Brain, Cognition and Behavior; Radboud university medical center, Nijmegen, the Netherlands

^6^Department of Neurobiology, Max Planck Institute for Biophysical Chemistry, Göttingen, Germany.

^7^La Trobe Institute for Molecular Science 1, La Trobe University, Melbourne, Australia.

^8^Cortical Structure & Function Group, Netherlands Institute for Neuroscience, Amsterdam, the Netherlands.

*** Correspondence:** Louie van de Lagemaat, Centre for Neuroregeneration and Centre for Clinical Brain Sciences, Chancellor’s Building, 49 Little France Crescent, Edinburgh EH16 4SB, United Kingdom

louie.van.de.lagemaat@ed.ac.uk

1. **Supplementary Figures and Tables**


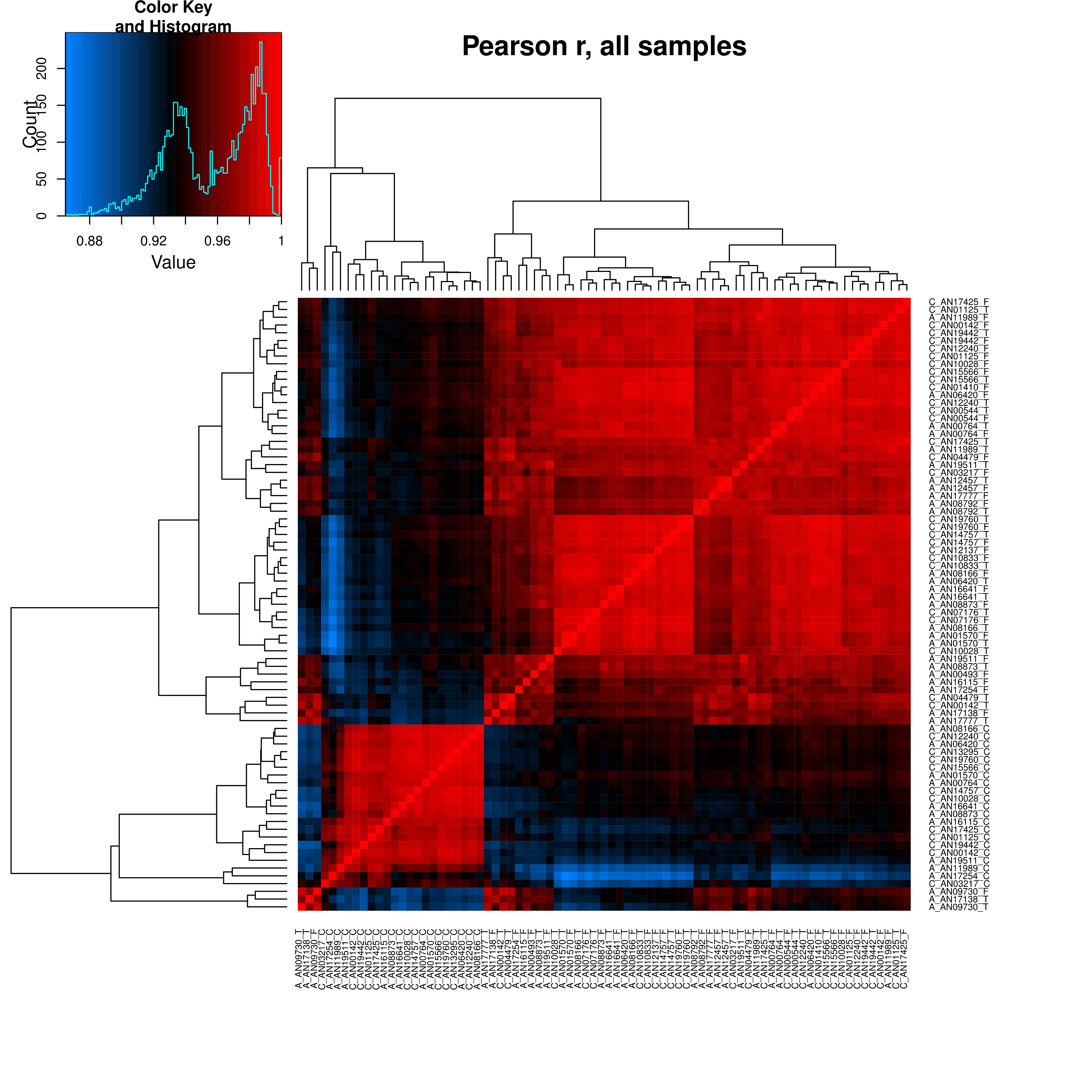


Supplementary Figure 1. Sample correlations (Pearson *r*) of original samples in the Voineagu dataset. Pairwise sample correlations are computed across expression values of all probes in each pair of samples. Sample labels are given as C or A for control or autism as a prefix, followed by the sample name, and then F, T, or C as a suffix (frontal cortex, temporal cortex, or cerebellum, respectively). Note that cerebellar samples cluster strongly, as do cortical samples. However, cortical samples do not segregate by frontal or temporal location.


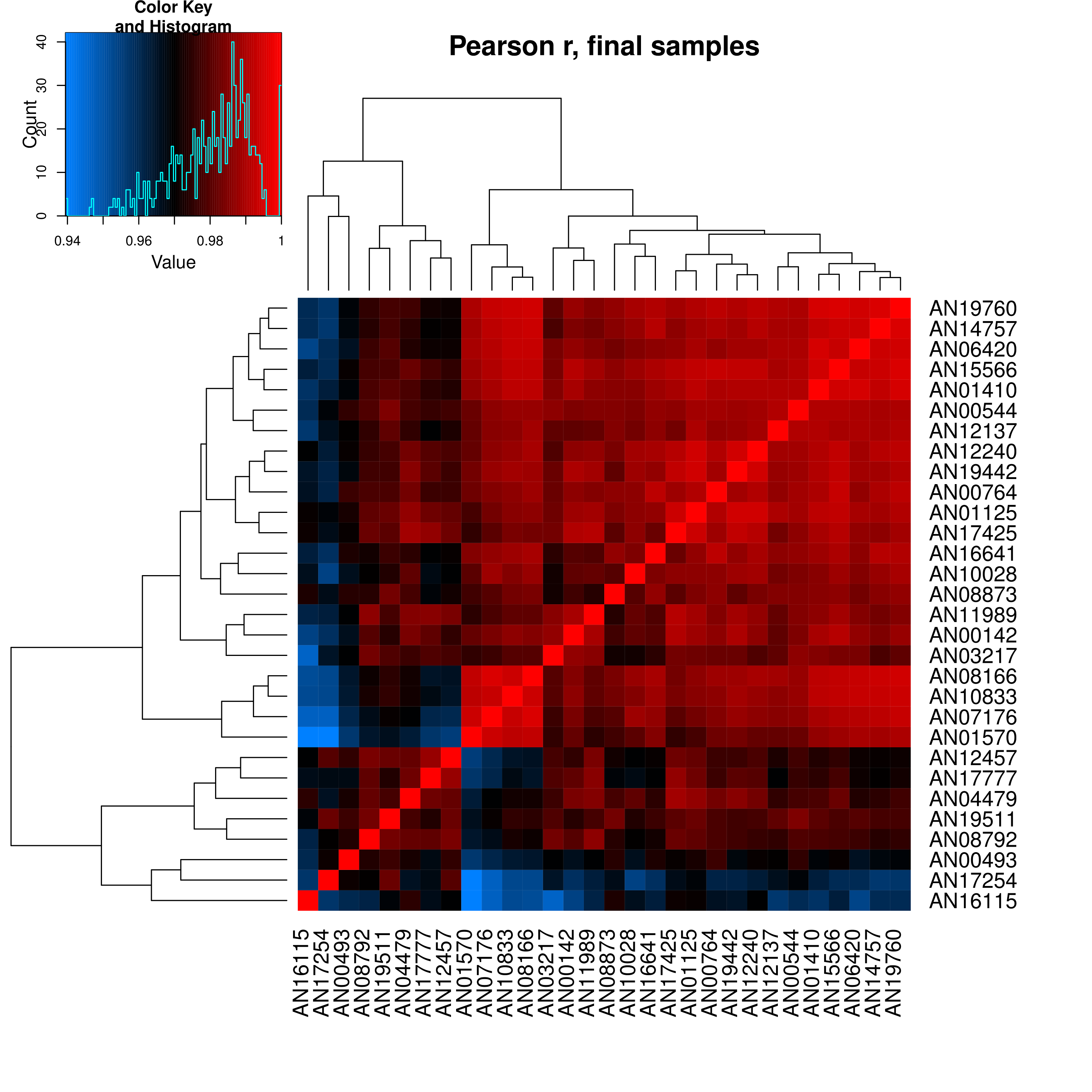


**Supplementary Figure 2. Correlations (Pearson *r*) of final cortical data from Voineagu dataset**. Multiple samples collapsed within an individual. Data are highly correlated, with *r* ≥ 0.94.


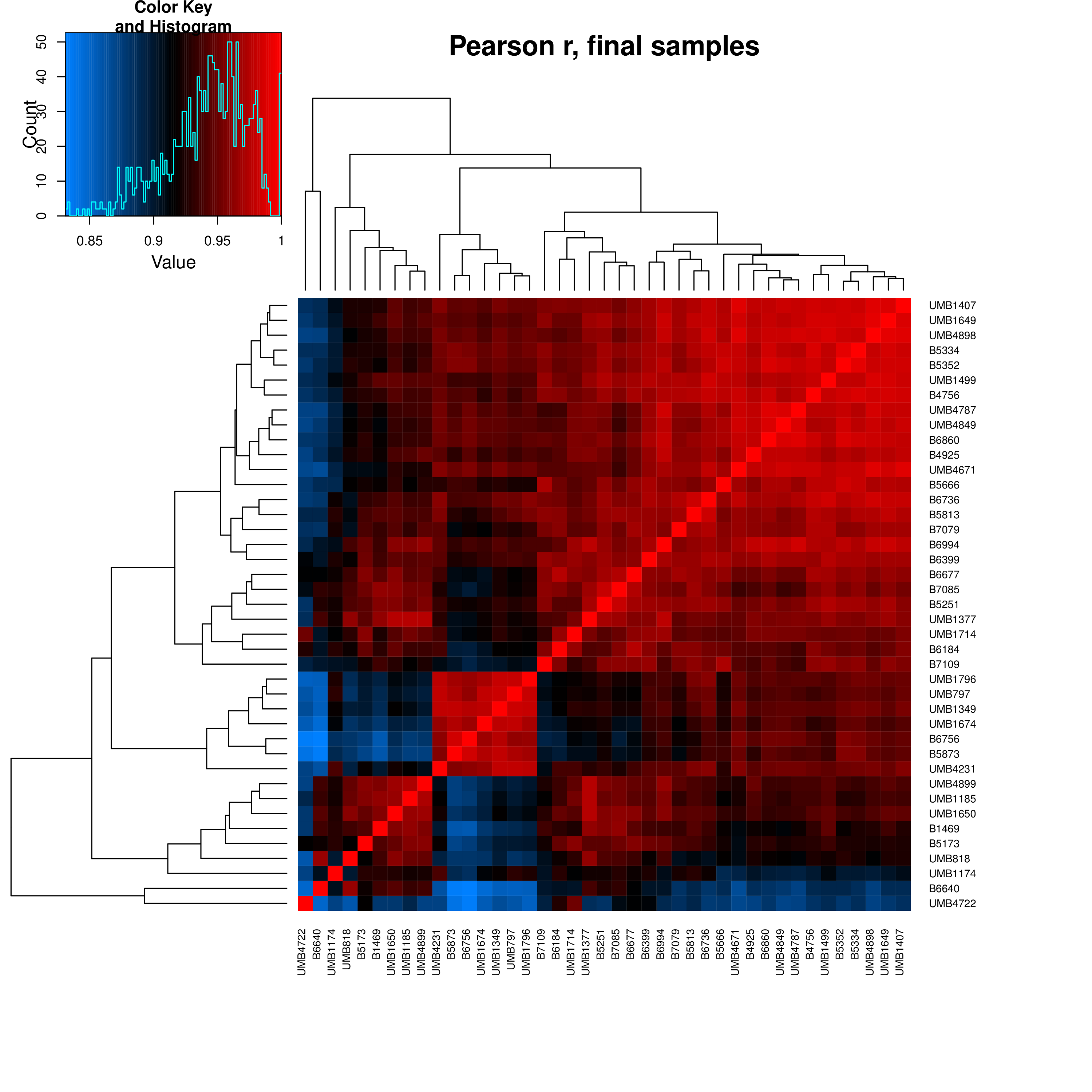


**Supplementary Figure 3. Correlations (Pearson *r*) of prefrontal cortical samples from Chow dataset**. Multiple samples collapsed within an individual. Correlation computed across all genes. Data are highly correlated, with *r* ≥ 0.82.


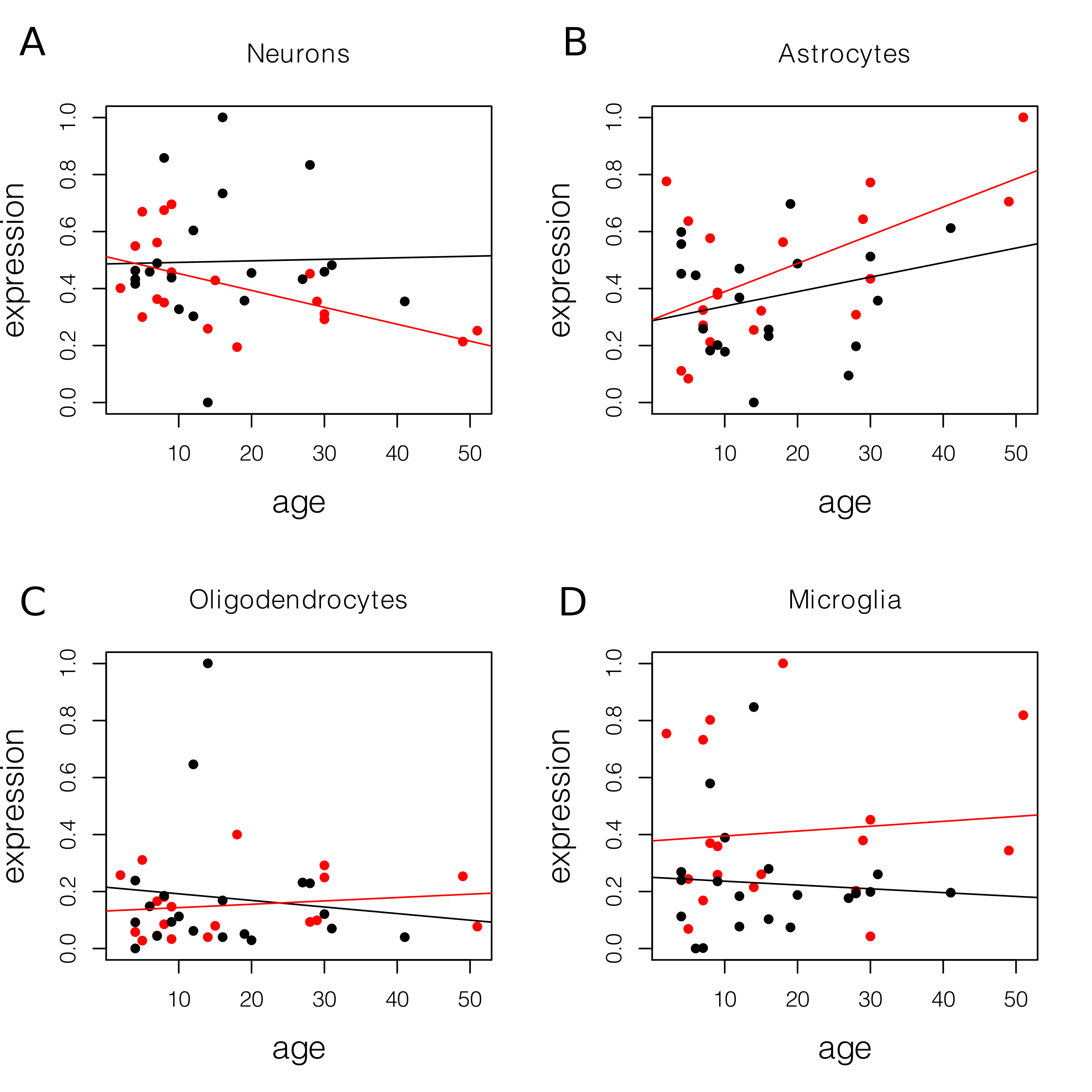


**Supplementary Figure 4. Change of expression of sets of nervous system cell-type markers in the Chow dataset**. Marker gene symbols, probe IDs, and model parameters are given in the table below. A. Neuronal markers. B. Astrocyte markers. C. Oligodendrocyte markers. D. Microglial markers.

**Supplementary Table 1. Age related changes in nervous system cell populations in autistic and control samples.**

| Marker type | Gene symbols [Probe IDs] | Model type | Model equation |
| --- | --- | --- | --- |
| Neurons | NEFL [ILMN_1659086],  ENO2 [ILMN_1765796], SLC12A5 [ILMN_2129910], KCNQ2 [ILMN_2300894], SCN3A [ILMN_1657591,  ILMN_2387395] | Controls | 0.48[±0.06] + 0.00054[±0.00259]*age |
|  |  | Case-control  difference | 0.026[±0.091] – 0.065[±0.004]*age |
| Astrocytes | GFAP [ILMN_1697176],  AQP4 [ILMN_1747683],  GJA1 [ILMN_1727087] | Controls | 0.29[±0.07] + 0.0051[±0.0028]*age |
|  |  | Case-control  difference | 0.0030[±0.1000] + 0.0048[±0.0043]*age |
| Oligo-dendrocytes | MOG [ILMN_2310001],  MAG [ILMN_2380181,  ILMN_1803773],  MOBP [ILMN_1768947,  ILMN_2414962],  MBP [ILMN_2331544,  ILMN_2398939] | Controls | 0.22[±0.06] – 0.0023[±0.0026]*age |
|  |  | Case-control  difference | -0.083[±0.093] + 0.0035[±0.0040]*age |
| Microglia | CD37 [ILMN_2375825,  ILMN_1786176],  CD53 [ILMN_1662843,  ILMN_2413808] | Controls | 0.25[±0.08] – 0.0013[±0.0034]*age |
|  |  | Case-control  difference | 0.13[±0.12] + 0.0030[±0.0051]*age |
